# Anatomical variations of the dentate gyrus in normal adult brain

**DOI:** 10.1007/s00276-019-02298-5

**Published:** 2019-08-01

**Authors:** Robert Haładaj

**Affiliations:** Department of Normal and Clinical Anatomy, Interfaculty Chair of Anatomy and Histology, Medical University of Lodz, ul. Żeligowskiego 7/9, 90-752 Lodz, Poland

**Keywords:** Cerebral cortex, Dentate gyrus, Human brain, Temporal lobe, Hippocampal arteries, Hippocampal formation

## Abstract

Recent scientific papers indicate the clinical significance of the dentate gyrus. However, a detailed knowledge of the anatomical variations of this structure in normal adult brain is still lacking. An understanding of the variable morphology of the dentate gyrus may be important for diagnostic neuroimaging. Thus, the purpose of this macroscopic cadaveric study was to describe the anatomical variations of the dentate gyrus. Forty formalin-fixed human cerebral hemispheres, obtained from bodies of donors without the history of neuropathological diseases, were included in the study. The dentate gyrus was classified as well-developed, when it protruded completely from under the fimbria of the hippocampus. The gyrus was classified as underdeveloped, when it was covered by the fimbria of the hippocampus (but clearly visible at the coronal section of the hippocampal formation), while the hypoplastic gyrus was not visible macroscopically under the fimbria of the hippocampus. The well-developed type was observed in 27 cases (67.5%). The thickness of well-developed type of the dentate gyrus, measured between the fimbriodentate sulcus and hippocampal sulcus, varied from 2.74 to 5.21 mm (mean = 3.67 mm, median = 5.54 mm, SD 0.65 mm). In the next nine cases (22.5%), the dentate gyrus was underdeveloped. The thickness of underdeveloped type of the dentate gyrus varied from 1.75 to 2.37 mm (mean = 2.02 mm, median = 2.16 mm, SD 0.33 mm). In the remaining four cases (10%), the dentate gyrus was hypoplastic and could not be distinguished macroscopically. In all injected hemispheres, arterial supply of the dentate gyrus was provided by the branches of the posterior cerebral artery. Awareness of normal variations of the dentate gyrus may allow for better correlation of anatomical knowledge with radiological data and for use this knowledge to describe abnormal conditions.

## Introduction

The dentate gyrus is a part of the larger functional brain system called the hippocampal formation, along with the hippocampus proper (Ammon’s horn) and subiculum of the parahippocampal gyrus [1, 2, 6, 19]. This cortical region is strongly associated with cognitive function. Neurons in the dentate gyrus of animals and humans play an important role in learning and memory [7, 9, 23]. The dentate gyrus is also an area identified as neurogenic in adult rodents, monkeys and humans [11, 19, 26].

The dentate gyrus along with other parts of the hippocampal formation create a “banana-shaped structure” [2]. The hippocampus may be subdivided in four different fields (CA1-CA4). The dentate gyrus contains the CA4 field [6]. Macroscopically, the dentate gyrus consists of small cortical prominences (the dentes of the dentate gyrus) located along the medial, concave margin of the hippocampus [22, 24]. The superficial part of the dentate gyrus, known as the margo denticulatus, is only visible at the medial, extra-ventricular aspect of the hippocampal body [6, 8]. The rounded protrusions (dentes) forming margo denticulatus are the largest in the middle and diminish in size caudally and cranially [8].

The dentate gyrus is separated from the fimbria of the hippocampus by the fimbriodentate sulcus, while a shallow groove called the hippocampal sulcus separates this gyrus from the subiculum of the parahippocampal gyrus [22]. Histologically, the dentate gyrus consists of three distinct layers: molecular, granular, and polymorphic. Functionally, the it plays a role of the gateway to the hippocampus. Jonas and Lisman [15] stress, that strong evidence suggests that the dentate gyrus acts as a preprocessor of incoming information, preparing it for subsequent processing in CA3 region. Structure, function, and plasticity of hippocampal dentate gyrus microcircuits have been the subject of numerous studies in recent times [2, 15, 17, 21].

The gyral organization in normal human brain may vary between individuals. Thus, an understanding of the anatomical variations in the human cortex may be important for neuroanatomy teaching, diagnostic neuroimaging and for neurosurgical procedures [10, 12, 13, 16, 18, 27, 28]. Anatomy of the hippocampal region is thought to be very complex and difficult to understand for non-specialized neurosurgeons or neurologists [6]. Thus, the purpose of this macroscopic cadaveric study was to describe the anatomical variations in morphology of the dentate gyrus. Awareness of normal variations of this structure may also allow for better correlation of anatomical knowledge with radiological data and for use this knowledge to describe abnormal conditions.

## Materials and methods

Forty formalin-fixed human cerebral hemispheres with no visible pathologies, obtained from bodies of donors without the history of neurological diseases in their clinical records, were included in the study. The hemispheres were fixed in 10% formalin and preserved in 70% ethanol solution. The research project was approved by the local Bioethics Committee.

**Fig. 1:**
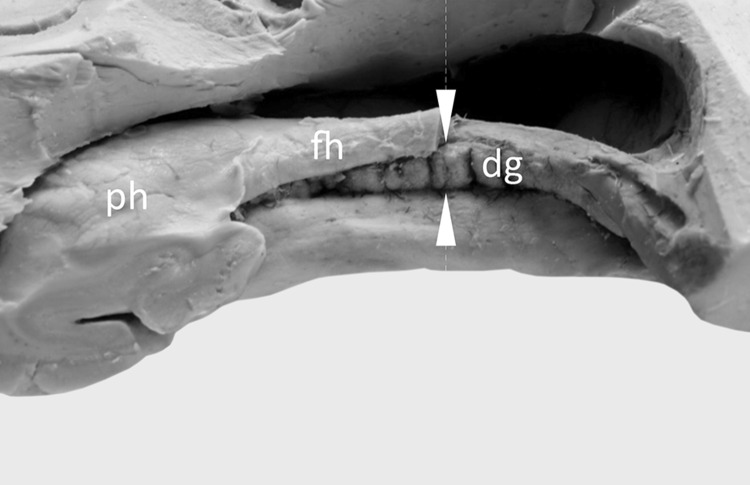
Assessment of the dentate gyrus morphology. The well-developed margo denticulatus of the dentate gyrus (dg) was visualized on the medial side of the fimbria of the hippocampus (fh). The tooth-shaped part of the dentate gyrus is clearly visible. For comparison purposes, horizontal incisions through each hippocampal formation were performed with the aid of a brain knife, in the half-length of the body of the hippocampus (place of the incision was marked by white arrowheads). At this level the thickness of the margo denticulatus was also measured, between the fimbriodentate sulcus and hippocampal sulcus. *ph* pes of the hippocampus

To expose the dentate gyrus, the temporal lobes were harvested. The inferior (temporal) horn of the lateral ventricle was opened. To that end, the roof of the inferior horn was removed and the hippocampus with its fimbria was exposed. On the medial side of the fimbria, the dentate gyrus (margo denticulatus) was visualized. Additionally, for comparison purposes, horizontal incisions through each hippocampal formation were performed with the aid of a brain knife, in the half-length of the body of the hippocampus (Fig. 1), because the margo denticulatus is best developed in this area [8]. The thickness of the dentate gyrus was assessed and measured at this level. The measurements were performed between the fimbriodentate sulcus and hippocampal sulcus (Fig. 1). Five hemispheres were injected by colored resin. That allowed to trace general pattern of the arterial supply of the dentate gyrus.

The preliminary classification of the dentate gyri was performed in situ. To verify the rating system, photographic documentation of specimens was obtained, according to previously described protocol [27]. Photographs were processed with MultiScanBase v.18.03 software (Computer Scanning System II, Warsaw, Poland) in order to recheck the assessment of each specimen. Due to lack of any uniform criteria for evaluation of the morphology of the dentate gyrus, own classification was introduced. The study used a simple visual classification of this gyrus, based on morphology of the margo denticulatus. The dentate gyrus was classified as well-developed, when the margo denticulatus protruded completely from under the fimbria of the hippocampus. In those cases, the hippocampal sulcus was well-developed and clearly visible along all length of the margo denticulatus. The gyrus was classified as underdeveloped, when the margo denticulatus was covered by the fimbria of the hippocampus (but clearly visible at the coronal section of the hippocampal formation), while a hypoplastic gyrus was recognized when the margo denticulatus was not visible macroscopically under the fimbria of the hippocampus. The degree of development of each variant of dentate gyri was confirmed by analyzing coronal sections of the hippocampal formation.

## Results

**Fig. 2:**
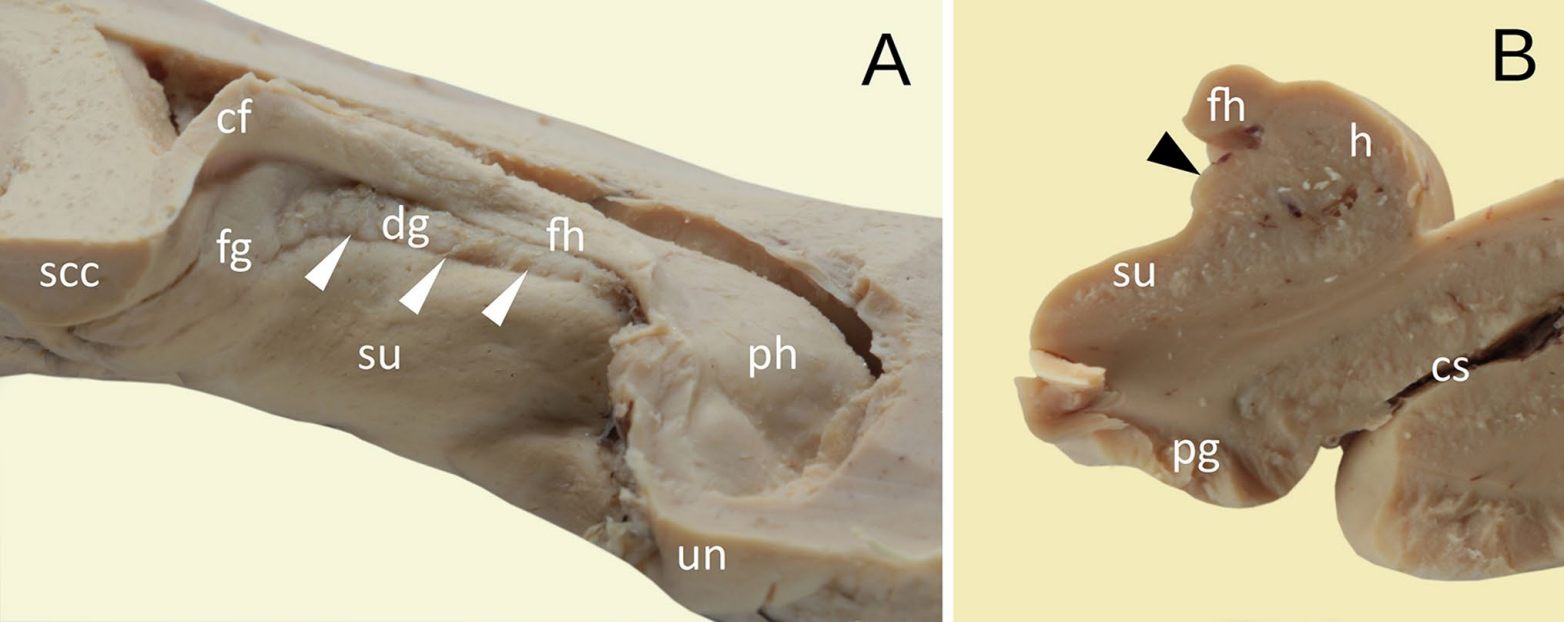
The appearance of the well-developed dentate gyrus. **a** Medial view. The dentate gyrus (dg) was classified as well-developed, when the margo denticulatus protruded completely from under the fimbria of the hippocampus (fh). In those cases, the hippocampal sulcus (marked by white arrowheads) was well-developed and clearly visible along all length of the margo denticulatus. **b** The coronal section through the hippocampal formation showing the well-developed dentate gyrus (marked by black arrowhead). *cf* crus of fornix, *cs* collateral sulcus, *fg* fasciolar gyrus, *h* hippocampus proper (Ammon’s horn), *pg* parahippocampal gyrus, *ph* pes of hippocampus, *scc* splenium of the corpus callosum, *su* subiculum of the parahippocampal gyrus, *un* uncus

**Fig. 3:**
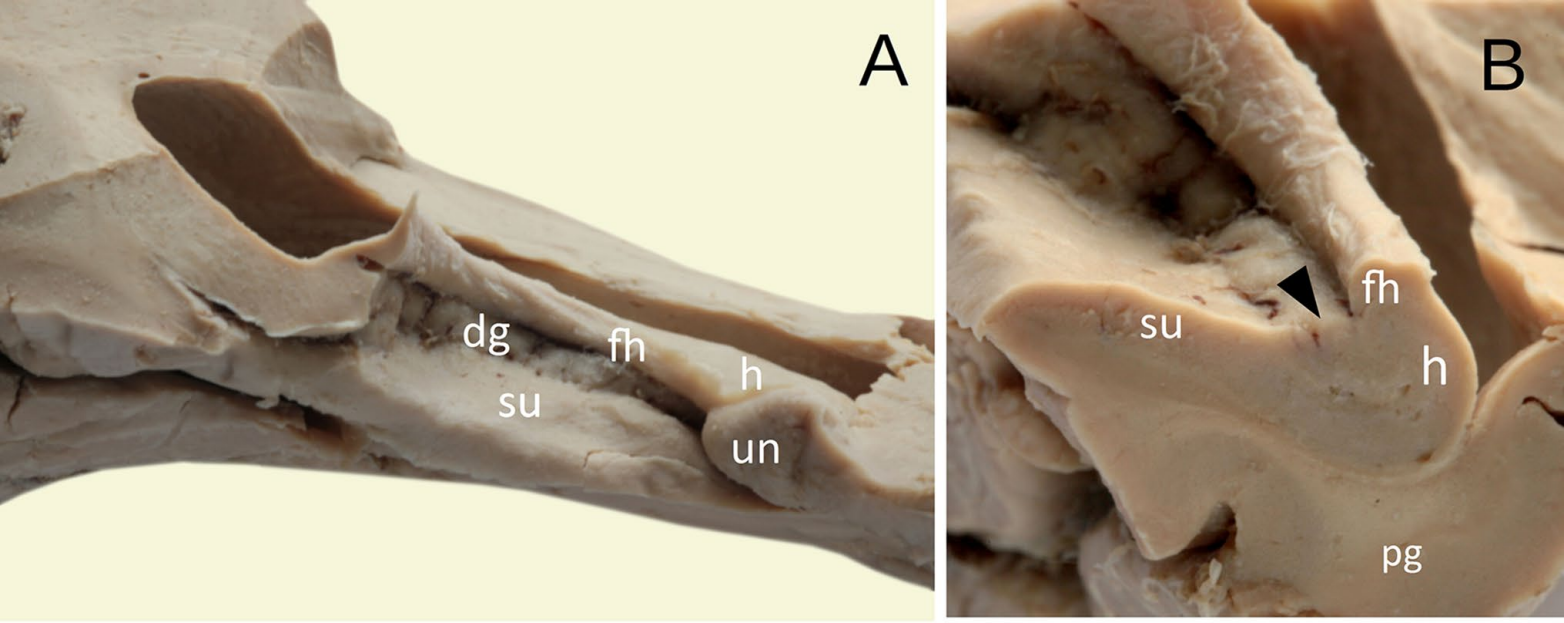
The appearance of the well-developed dentate gyrus with irregular prominences and less marked hippocampal sulcus. **a** Medial view. **b** The coronal section through the hippocampal formation showing the dentate gyrus (marked by black arrowhead). *h* hippocampus proper (Ammon’s horn), *dg* margo denticulatus of the dentate gyrus, *fh* fimbria of the hippocampus, *pg* parahippocampal gyrus, *ph* pes of hippocampus, *su* subiculum of the parahippocampal gyrus

Three basic types of dentate gyrus have been distinguished on the examined material. The well-developed type was observed in 27 cases (67.5%). Including 14 cases, the dentate gyrus consisted of numerous regular prominences separated from each other by distinct vascular branches (Figs. 1, 2). The width of the prominences in this type of the dentate gyrus varied from 1.72 to 4.01 mm (mean = 2.37 mm, median = 2.08 mm, SD 0.63 mm). The thickness of margo denticulatus of well-developed type of the dentate gyrus, measured between the fimbriodentate sulcus and hippocampal sulcus, varied from 2.74 to 5.21 mm (mean = 3.67 mm, median = 5.54 mm, SD = 0.65 mm). In those cases, the hippocampal sulcus was clearly visible along the margo denticulatus. In 13 cases out of those representing well-developed type of the dentate gyrus, the prominences were irregular, and the hippocampal sulcus was less marked (Fig. 3).

**Fig. 4:**
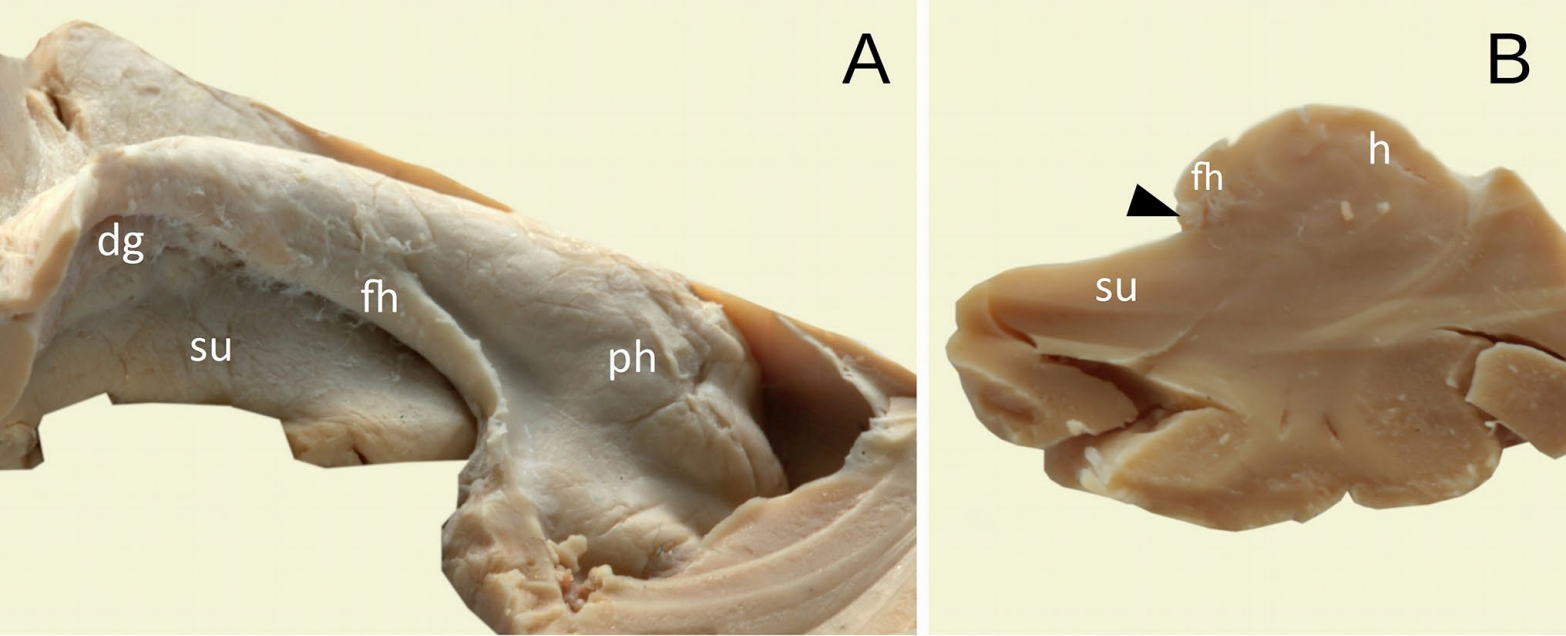
The appearance of the underdeveloped dentate gyrus. **a** Medial view. The dentate gyrus was classified as underdeveloped, when the margo denticulatus (dg) was covered by the fimbria of the hippocampus (fh). **b** The coronal section through the hippocampal formation showing the underdeveloped margo denticulatus (marked by black arrowhead). *h* hippocampus proper (Ammon’s horn), *ph* pes of hippocampus, *su* subiculum of the parahippocampal gyrus

**Fig. 5:**
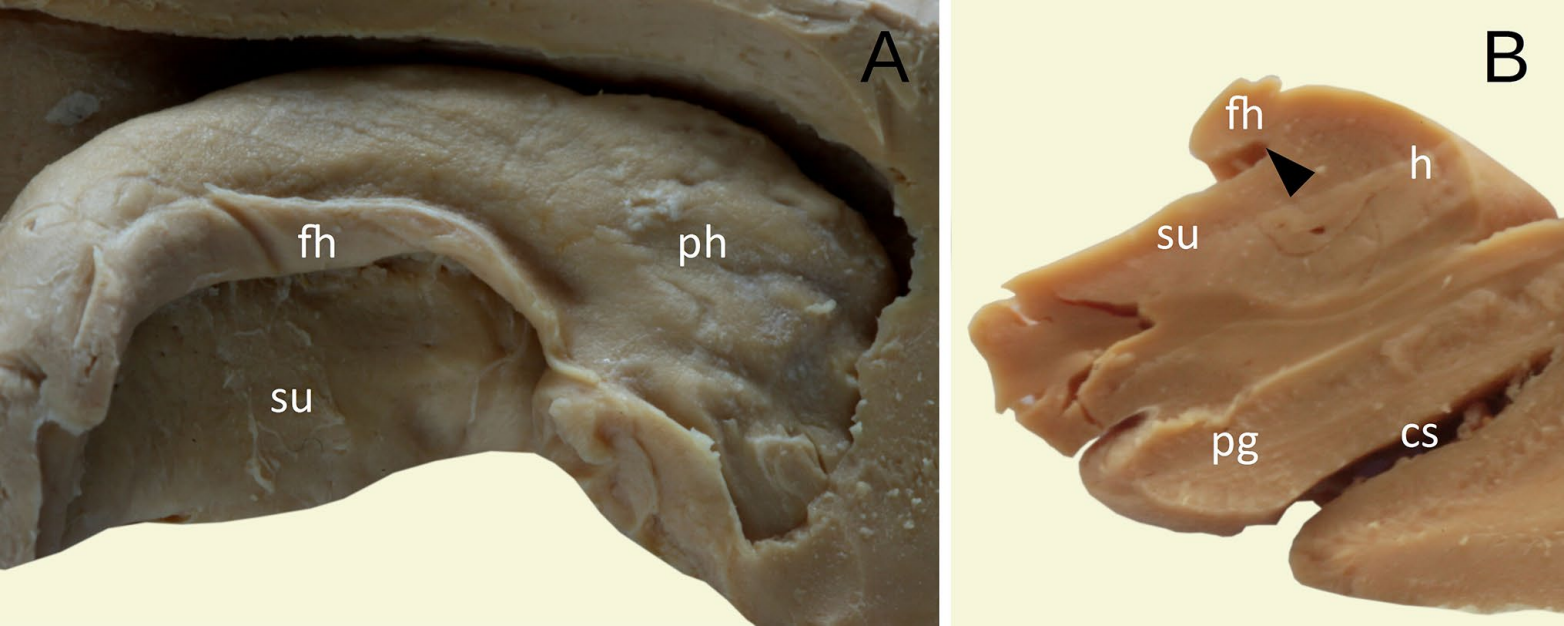
Hypoplasia of the dentate gyrus. **a** Medial view. The dentate gyrus was classified as underdeveloped, when the margo denticulatus was not visible macroscopically under the fimbria of the hippocampus (fh). **b** The coronal section through the hippocampal formation showing the hypoplasia of the dentate gyrus (black arrowhead shows the place with absent or vestigial margo denticulatus of dentate gyrus). *cs* collateral sulcus, *h* hippocampus proper (Ammon’s horn), *pg* parahippocampal gyrus, *ph* pes of hippocampus, *su* subiculum of the parahippocampal gyrus

In the next nine cases (22.5%), dentate gyrus was underdeveloped. In those cases, the margo denticulatus was covered by the fimbria of the hippocampus, but clearly visible at the coronal section of the hippocampal formation (Fig. 4). The width of the prominences in this type varied from mm 1.01 to 3.02 mm (mean = 1.82 mm, median = 1.58 mm, SD 0.6 mm). The thickness of margo denticulatus of underdeveloped type of the dentate gyrus, measured between the fimbriodentate sulcus and hippocampal sulcus, varied from 1.75 to 2.37 mm (mean = 2.02 mm, median = 2.16 mm, 0.33 mm). Regardless of the type of the dentate gyrus, the width and height of the prominences on margo denticulatus reached the highest values at the level of the middle of the length of the hippocampal body, gradually reducing their dimensions in the caudal direction. In the remaining four cases, the dentate gyrus was hypoplastic and could not be distinguished macroscopically (Fig. 5).

**Fig. 6:**
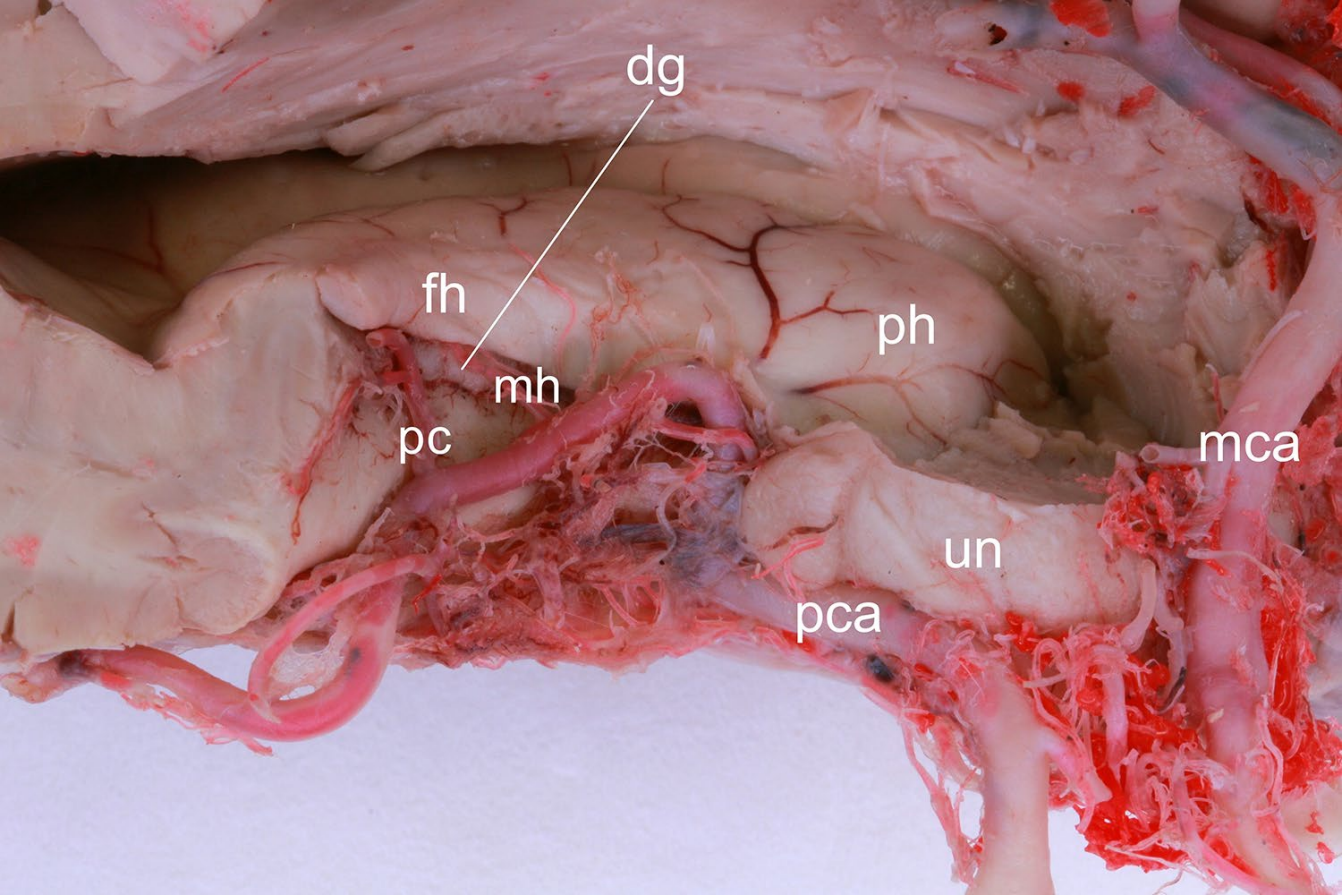
Arterial supply of the dentate gyrus. Arterial supply of the dentate gyrus is provided by the branches of the posterior cerebral artery (pca), namely by one of the posterior choroidal arteries (pc) and by the middle hippocampal artery (mh). *dg* margo denticulatus of the dentate gyrus, *fh* fimbria of the hippocampus, *h* hippocampus proper (Ammon’s horn), *mca* middle cerebral artery, *ph* pes of hippocampus, *un* uncus

In all injected hemispheres, arterial supply of the dentate gyrus was provided by the branches of the posterior cerebral artery, namely by some of the posterior choroidal arteries and by the middle hippocampal artery (Fig. 6).

## Discussion

According to Pamar et al. [19], a detailed knowledge of the anatomical variations and symmetry pattern of the medial temporal structures, including dentate gyrus, is still lacking. Most of the dentate gyrus is not exposed onto the brain surface. Swanson [24] describes the dentate gyrus as the most medial component of the hippocampal region, located adjacent to the Ammon’s horn. However, there are inaccuracies in the literature regarding the anatomical description of this structure. The term dentate gyrus was used by Honegger in 1890 and Ranson in 1920 [24]. Swanson [24] gives also other examples of synonyms for the term “dentate gyrus”. It was first delineated as “substantia cinerea” by Tarin in 1750, Vicq d’Azyr referred it as “internal dentate margin of Ammon’s horn”, “internal (concave, dentate or gadroonate) margin of hippocampus major” or “crenate part of hippocampus major”. Reil, in turn, used the term “indented border”, Döllinger referred the dentate gyrus as “fascia dentata”, while Volkmann described the dentate gyrus as “taeniam cornu Ammonis cineream” [24].

In human, the hippocampus gets a complex rolled structure due to its rotation along the longitudinal axis. According to description of Destrieux et al. [6] the hippocampus is C-shaped and made of two rolled-up laminae: the cornu Ammonis (hippocampus proper) and the gyrus dentatus. The gyrus dentatus forms a dorso-medially concave structure, limited by the fimbrio-dantate sulcus and hippocampal sulcus. Destrieux et al. [6] emphasize that some terminological inaccuracies may arise from the fact that the dentate gyrus can have different names, depending on the part of the hippocampus with which it is adjacent. It appears as the “margo denticulatus” at the level of body of the hippocampus, as the “medial band of Giacomini” at the medial aspect of the uncus, and as the “fasciola cinerea” at the level of the tail of hippocampus.

Because the dentate gyrus shows trilaminar morphology, it is classified (similarly to other hippocampal areas) as archicortex. The structure of mammalian dentate gyrus may be compared to reptile and bird medial cortex, in the context of evolutionary relationships [14]. Hevner et al. [14] points also to the fact that neocortical gyrification appears to have evolved at about the same time as convolution of the dentate gyrus in stem mammals. Available data indicate that dentate gyrus traits are present in all orders of mammals, except for whales, dolphins, and porpoises, in which this gyrus size, convolution, and adult neurogenesis undergone evolutionary regression [14]. In acallosal mammals, the hippocampus is organized around a longitudinal hippocampal sulcus that invaginates at the medial wall of the hemisphere and remains clearly visible in the adult. In Humans, hippocampal sulcus is less developed but also present at the medial aspect of the temporal lobe [6]. The tooth-shaped part of the dentate gyrus, known as the margo denticulate, is visible at the medial, extra-ventricular aspect of the hippocampal body. The presence of dentes on the margo denticulatus is specific to humans and higher primates [8].

Most of the arterial supply of the hippocampal formation from the posterior cerebral and postero-lateral choroidal arteries [25]. In our study we observed, that arterial supply of the dentate gyrus came specifically from branches of the posterior cerebral artery, namely from the middle hippocampal artery and selected posterior choroidal arteries. Duvernoy [8], analyzing research on vascularization of the hippocampus, summarizes that three arteries (or groups of arteries) supply the hippocampus, namely: the anterior, middle and posterior hippocampal arteries. The middle and posterior hippocampal arteries, after reaching the hippocampus, follow the course parallel to margo denticulatus and give rise to two kinds (small and large) of branches. The large branches (“straight arteries”) reach the sulci between the dentes of the dentate gyrus, while the small branches penetrate the whole surface of the margo denticulatus [8]. Duvernoy hypothesizes that penetration of the large arteries on the surface of margo denticulatus may contribute to formation of characteristic denticulations [8].

Comparison of the data on the morphology of the dentate gyrus obtained in the presented study is difficult due to only a few mentions of the gross anatomy of this structure which we were able to find in the literature. The study of Pamar et al. [19] is one of very few studies in which the degree of development of the dentate gyrus was determined. In the cited study the margo denticulatus part of dentate gyrus and fasciolar gyrus were well defined in 65.3% of cases. This data is consistent with presented study, in which well-developed dentate gyrus was found in 67.5% of examined cases. In turn, bilateral agenesis of the hippocampal dentate gyrus in a neurologically normal adult was described by Clark and Sarnat [4]. Those authors concluded, based on medical history and detailed microscopic examination (including appropriate histochemical and immunohistochemical preparations) of the brain of neurologically normal 82-year-old man, that bilateral agenesis of the hippocampal dentate gyrus, and apparent failure of regeneration of dentate granule cells from stem cells in adult life, may occur without overt clinical neurological deficits [4]. This observation may suggest, that agenesis (defined in the presented study as hypotrophy) may potentially be anatomical variant of hippocampal formation morphology. However detailed neurological and neuropsychological evidences are needed to confirm such an assumption.

Current scientific studies prove the clinical significance of the dentate gyrus. In study of Redwine et al. [20], high-resolution magnetic resonance microscopy was used to determine regional brain volumetric changes in a mouse model of Alzheimer’s disease. In this study the dentate gyrus volume was reduced before onset of plaque formation. Elvsåshagen et al. [10] demonstrated, that decreased volume of the dentate gyrus was associated with depressive episodes. Longoni et al. [18] studied correlation between physical activity and dentate gyrus volume in pediatric acquired demyelinating syndromes. According to the findings of this study, the dentate gyrus volume in pediatric patients with acquired demyelinating syndrome was greater in children who have recovered from monophasic demyelination. Hayes et al. [12] suggest that dentate gyrus abnormalities are associated with symptoms of posttraumatic stress disorder. Daughtery et al. [5] suggest in turn, that hippocampal CA3-dentate gyrus volume uniquely linked to improvement in associative memory from childhood to adulthood. Boldrini et al. [3] suggest, based on postmortem examination, that resilience to early life adversity is associated with larger dentate gyrus, while suicide decedents with major depressive disorder have fewer granule neurons, as well as less neurogenesis and/or more apoptosis and dendrite changes in this region.

## Limitations of the study

Although major pathologies have not been observed on the material examined in the presented work, the combination of clinical studies with accurate imaging diagnostics can complement the scope of anatomical knowledge with fully reliable data. Especially, the issue of hypoplasia of the dentate gyrus needs further explanation based on clinal and neuroimaging assessment. The presented work was carried out on isolated hemispheres, and therefore it was not possible to obtain data on the symmetry or asymmetry of the dentate gyrus between the brain hemispheres. However, this report provides easy and useful visual criteria for the assessment of the morphology of the dentate gyrus.

## Conclusions

The anatomical knowledge may still play a key role in modern neuroimaging analysis of brain structure and function in the era of advances in noninvasive imaging methods. Awareness of normal variations of the dentate gyrus may allow for better correlation of anatomical knowledge with radiological data and for use this knowledge to describe abnormal conditions.
